# Copper trafficking in eukaryotic systems: current knowledge from experimental and computational efforts

**DOI:** 10.1016/j.sbi.2019.05.002

**Published:** 2019-10

**Authors:** Alessandra Magistrato, Matic Pavlin, Zena Qasem, Sharon Ruthstein

**Affiliations:** 1 National Research Council of Italy-IOM c/o International School for Advanced Studies (SISSA), via Bonomea 165, 34135, Trieste, Italy; 2 The Chemistry Department, Faculty of Exact Sciences, Bar-Ilan University, 529002, Israel

## Abstract

Copper plays a vital role in fundamental cellular functions, and its concentration in the cell must be tightly regulated, as dysfunction of copper homeostasis is linked to severe neurological diseases and cancer. This review provides a compendium of current knowledge regarding the mechanism of copper transfer from the blood system to the Golgi apparatus; this mechanism involves the copper transporter hCtr1, the metallochaperone Atox1, and the ATPases ATP7A/B. We discuss key insights regarding the structural and functional properties of the hCtr1-Atox1-ATP7B cycle, obtained from diverse studies relying on distinct yet complementary biophysical, biochemical, and computational methods. We further address the mechanistic aspects of the cycle that continue to remain elusive. These knowledge gaps must be filled in order to be able to harness our understanding of copper transfer to develop therapeutic approaches with the capacity to modulate copper metabolism.

## Highlights

• The main copper transporter, Ctr1, can transfer Cu(I) in the cell, through two different intracellular domains.
• Conformational flexibility of the copper metallochaperone Atox1 controls copper transfer mechanism in the cell.
• Each metal binding domain in ATP7B has a specific role.

## Graphical abstract

**Figure:**
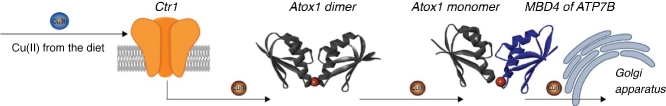

**Current Opinion in Structural Biology** 2019, **58**:26–33

This review comes from a themed issue on **Biophysical and computational methods**

Edited by **Alan R Lowe** and **Laura S Itzhaki**

For a complete overview see the Issue and the Editorial

Available online 6th June 2019

**https://doi.org/10.1016/j.sbi.2019.05.002**

0959-440X/© 2019 The Authors. Published by Elsevier Ltd. This is an open access article under the CC BY license (http://creativecommons.org/licenses/by/4.0/).

## Introduction

Copper, like other metals, has a pivotal role in fundamental processes of cell function. It takes part in cellular respiration, iron oxidation, pigment formation, neurotransmitter biosynthesis, antioxidant defense, and connective tissue formation. Yet, when present at excessive concentrations, it can endanger the cell’s survival, by causing de-regulated oxidation of proteins, lipids, and other cellular components, ultimately leading to injury. Moreover, free Cu ions can produce radical oxygen species (ROS), which can lead to cytotoxic interactions with cell membranes [1, 2,3^•^,^4^]. Insufficient concentrations of copper, in turn, can lead to metabolic abnormalities, as copper-dependent proteins drive iron absorption, and a copper deficiency can therefore lead to iron deficiency. Thus, intracellular pathways of copper metabolism have evolved to ensure the appropriate amount of Cu for cell survival.

**Figure 1:**
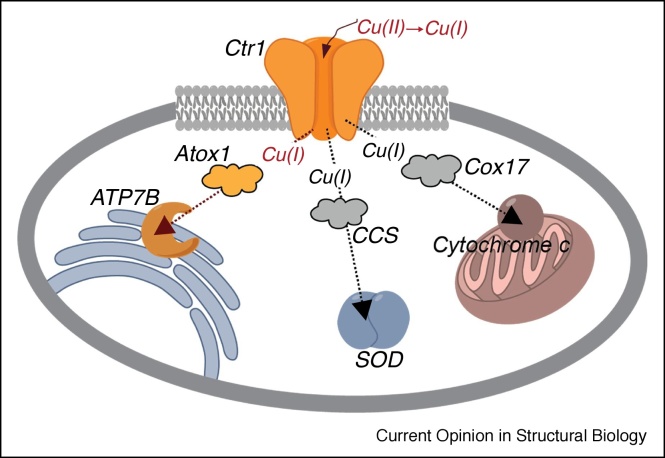
The human copper cycle system.

Broadly, copper follows the following trajectory through the human body: First, it accumulates in the blood through diet. Once it has been ingested, it is taken up from the blood by the copper transporter hCtr1. The copper is then reduced from its oxidized form, Cu(II), to the Cu(I) form; the mechanism of reduction is not fully known, as elaborated in what follows. Then, the transporter translocates the Cu(I) into the cell. Next, specific Cu(I) chaperones deliver the metal to the appropriate cellular pathways (Figure 1) [5, 6, 7, 8]. One such chaperone is Atox1, which transfers Cu(I) to its transporting ATPases in the Golgi apparatus. These ATPases include ATP7A and ATP7B, which play a biosynthetic role, delivering Cu(I) to the secretory pathway for metalation of cuproenzymes, and a homeostatic role, exporting excess Cu(I) from the cell. Additional chaperones include CCS, which is required for Cu(I) incorporation into cytoplasmic Cu/Zn superoxide dismutase, and Cox17, which delivers Cu(I) to mitochondrial cytochrome c oxidase.

In what follows, we provide an overview of current knowledge regarding the hCtr1-Atox1-ATP7B cycle. The hCtr1-Atox1-ATP7A/B cycle is associated with Menkes’ disease and with Wilson’s disease, in which mutations in ATP7A/B disrupt the homeostatic copper balance, resulting in Cu deficiency or overload, respectively. Beside these rare genetic diseases, the cycle has been implicated in Alzheimer‘s and Parkinson‘s diseases, and in anti-cancer drug resistance [1,2,9, 10, 11, 12]. Here, we summarize atomic-level type of information on key residues that are significant for function, and structural and kinetic insights on the copper transfer mechanism along its delivery path from the blood system to the Golgi apparatus and/or its egress from the cell.

## Experimental and computational approaches used to study copper trafficking

This review compiles information obtained through diverse experimental and computational methodologies. These latter include X-ray crystallography and nuclear magnetic resonance (NMR) spectroscopy, which were used to solve the 3D structures of the chaperone Atox1 [13^••^] and the metal-binding domains (MBDs) of ATP7A/B [14, 15, 16]. Single molecule FRET (sm-FRET) was used to obtain kinetic information on Cu(I) transfer from Atox1 to ATP7B [17^••^]. Electron paramagnetic resonance (EPR) spectroscopy has been used to characterize the various conformational states of Atox1 in solution while interacting with its partner proteins [18]. Moreover, ultraviolet-visible spectroscopy (UV-VIS) experiments and cell experiments were performed to target essential residues for Cu coordination and function. Computational methods such as molecular dynamics (MD) simulations, relying on force fields, have been applied to explore the free energy landscape of protein monomers and dimers mediating Cu transport [19], whereas mixed quantum classical simulations (QM/MM), able to overcome limitations of predefined force fields, have been employed to properly characterize metal Cu(I) coordination to these proteins, as well as its reaction/transfer mechanism [20].

## Copper uptake by the transporter hCtr1

**Figure 2:**
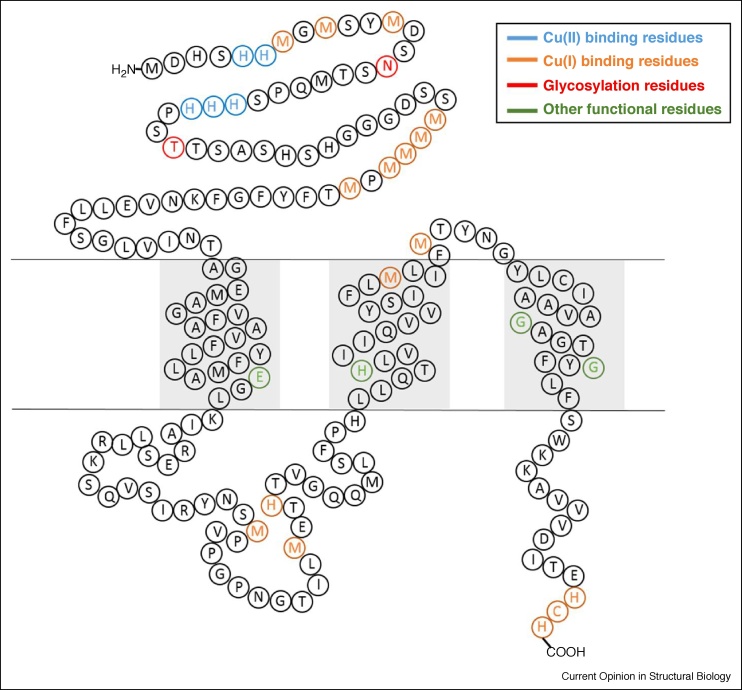
hCtr1 monomer sequence. Special residues are marked with distinct colors.

In 1997, Zhou and Gitschier became the first to identify a human gene for copper uptake, h*CTR1*. They showed that each hCtr1 polypeptide contains 190 amino acids [21]. Ten years later Unger *et al.* [22^•^,^23^] reported a three-dimensional, 6-Å-resolution structure of hCtr1 using cryogenic electron microscopy; they showed that the protein is a trimer containing: (1) 60 amino acids in the extracellular N-terminal domain; (2) three transmembrane (TM) helices (TM1, 2, and 3); (3) an intracellular loop of 46 amino acids, connecting TM1 and TM2; and (4) a short intracellular C-terminal domain with 15 amino acids (Figure 2).

*The extracellular domain of hCtr1* is characterized by several motifs: glycosylation sites, histidine (His)-rich sites, and methionine (Met) motifs. There are two glycosylation sites in hCtr1: N15 and T27. N15 is not required for function [24^•^]. Conversely, when O-glycosylation at T27 is prevented, the first 30 amino acids of hCtr1 are cleaved [25].

The His-rich sites in the extracellular domain of hCtr1—comprising two motifs, 1MDHxHH and ^22^HHH—are suggested to serve as Cu(II) binding sites. This idea is supported by UV-VIS experiments showing that the extracellular part of hCtr1 can bind two Cu(II) ions [26^••^]. EPR spectroscopy experiments suggest that the process by which Cu(II) is transferred from the blood to hCtr1 involves the blood carrier protein human serum albumin (HSA), as reflected in evidence of close interactions between HSA and the N-terminal domain of hCtr1 [27].

The Met-rich motifs in the extracellular domain—two segments, 7MxMxxM and 41MMMxM—are shared by many proteins involved in Cu(I)-metabolism; these segments bind Cu(I) with μM affinity [28], and they are essential for hCtr1’s recruitment of Cu(I), produced through the reduction of Cu(II) [29,30^•^]. Du *et al.* used UV-VIS titrations to show that three Cu(I) ions can coordinate to the extracellular part of hCtr1 [26^••^]. A combination of EPR, NMR, and UV-VIS experiments performed on the first 14 residues of hCtr1 indicated that H5 and H6 form the first Cu(II) binding site, whereas M7, M9 and M12 constitute the first Cu(I) binding site [31].

Notably, the mechanism of the Cu(II)-to-Cu(I) reduction process remains unclear. It has been suggested that copper and iron metabolism are intimately linked, with the two metals mutually participating in each other’s oxidation/reduction reactions. This proposition is supported by observations that in the yeast *Saccharomyces cerevisiae*, both Cu(II) and Fe(III) are reduced in the plasma membrane by Fre1 or Fre2 [32,33], and both Cu(I) and Fe(II) are oxidized (to Cu(II) and to Fe(III), respectively) by Fet3 metalloxidase [34,35].

*The TM domain of hCtr1* is characterized by conserved ^150^MxxxM and ^167^GxxxG motifs. De Feo *et al.* identified M150 and M154 in TM2 as Cu(I)-binding residues, whereas G167 and G171, both located in TM3, were proposed to mediate a tight interface between TM1 and TM3 [23]. Schushan *et al.* constructed a Cα-trace model of the TM domain of hCtr1, which agreed well with the experimental structure [36^••^]. Using a Gaussian network model and an anisotropic network model, they identified possible functional motions of the TM helices. On the basis of these motions, the authors proposed a transport mechanism in which the Cu(I) ions are transferred one at a time, and M154 (which points to the extracellular part of the membrane) along with H139 and E84 (conserved residues) control the transporter’s motion as a function of metal ion binding and a pH shift. This model is supported by biochemical experiments showing that H139 and E84 are indeed important functional residues [37,38]. Tsigelny *et al.* used all-atom simulations to develop a model for the full structure of hCtr1 [39]; using a Cα trace approach, they suggested that M43, M45, M150, and M154, and the ^188^HCH motif in the C-terminal domain are important residues for Cu(I) binding.

*The intracellular domain of hCtr1* contains two parts: an intracellular loop between TM1 and TM2, and a short C-terminal tail. The last 15 residues, ending with a ^188^HCH motif, are essential for Cu(I) transport. Kaplan and co-workers used ^64^Cu cell experiments to show that C189 is not essential for Cu(I) uptake [24^•^], but that ^188^HCH residues are vital for transfer and regulation purposes [37]. In the intracellular domain C189 was found to be critical for Cu(I) binding and transfer to Atox1 [37,40^•^]. NMR experiments revealed that Cu(I) binds to ^188^HCH with high affinity (*K*_D_ of 10^−14^ M) [41]. As a result, Cu(I) can be released to its target by protein–protein interaction, while being unable to freely dissociate from hCtr1. A recent study that used EPR along with circular dichroism (CD) and NMR [42] showed that the intracellular loop can form a second low-affinity Cu(I)-binding site (*K*_D_ ∼1–10 μM) involving the residues M106, M117, and H120.

## Distribution of Cu(I) by the metallochaperone Atox1

**Figure 3:**
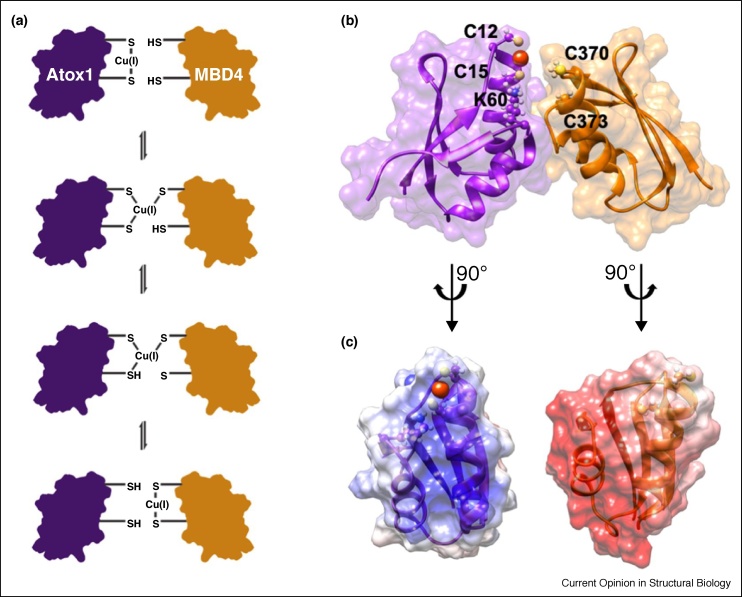
**(a)** Proposed mechanism for Cu(I) transfer from Atox1 to MBD4. **(b)** Representative structure obtained by MD for the interaction between holo-Atox1 and MBD4. **(c)** The structure and electrostatic potential surface of holo-Atox1 monomer and MBD4.

Atox1, also called Hah1, is a soluble protein (68 amino acids), displaying a βαββαβ fold [13^••^]. It coordinates one Cu(I) ion with the cysteine residues of a conserved 10MxCxxC motif (Figure 3). Cu(I)’s binding affinity toward Atox1 is even higher than that for the C-terminal domain of hCtr1 (*K*_D_ = 10^−17.4^ M), enabling Cu(I) to be transferred from hCtr1 to the metallochaperone [43].

Data obtained from the crystal structure of Atox1, as well as from NMR experiments and QM/MM simulations, point to additional residues, besides the conserved Cys-based motif that are important to Atox1 function. Specifically, K60 was found to be important for neutralizing the negative charge in the Cu(I) binding site, and T11 was shown to contribute to the Atox1’s flexibility [44, 45, 46]. Additionally, M10, completely conserved across different organisms, is buried in the hydrophobic core, contributing to Atox1’s stability [47]. Far-UV CD titration experiments in the pH range 6–11 showed that Cu(I) affinity for Atox1 decreases with the pH level, owing to protonation of the cysteine residues in the binding site [48^••^]. Considering that hypoxic malignant cells are characterized by low pH, this finding might point to a mechanism by which cancer interferes with Cu-homeostasis [49,50].

Notably, sm-FRET experiments have demonstrated that Atox1 can assume two conformational states, both able to interact with the target protein ATP7B [17^••^]. EPR measurements together with computations have resolved these two conformational states [18,40^•^,^42^], further suggesting that Atox1 can adopt conformations specific to its target protein. EPR experiments have also revealed the importance of ^188^HCH in stabilizing the complex comprising the C-terminal tail of hCtr1 and Atox1 [40^•^], and have confirmed that Atox1 interacts with the intracellular domain of hCtr1 (both the C-terminal tail and the intracellular loop) as a homodimer. The Atox1 homodimer has been solved by X-ray studies showing that the Atox1 dimer is stabilized by metal-mediated interactions, and by complementarity in the electrostatic interactions between the two monomers [44].

## Cu(I) uptake by the metal-binding domains of ATP7B

ATP7B is made of eight TM segments and two large cytosolic domains: (i) the N-terminal Cu-binding domain, and (ii) the catalytic ATP hydrolyzing domain [51]. Over 300 mutations in ATP7B have been identified that affect its function, indicating that almost one-third of the encoded residues are strictly required for proper ATP7B function. These observations suggest that, beyond identifying point mutations that affect Cu-homeostasis, it is necessary to obtain a detailed mechanistic picture of Cu(I) transfer in order to unravel the molecular basis of its metabolism.

**Figure 4:**
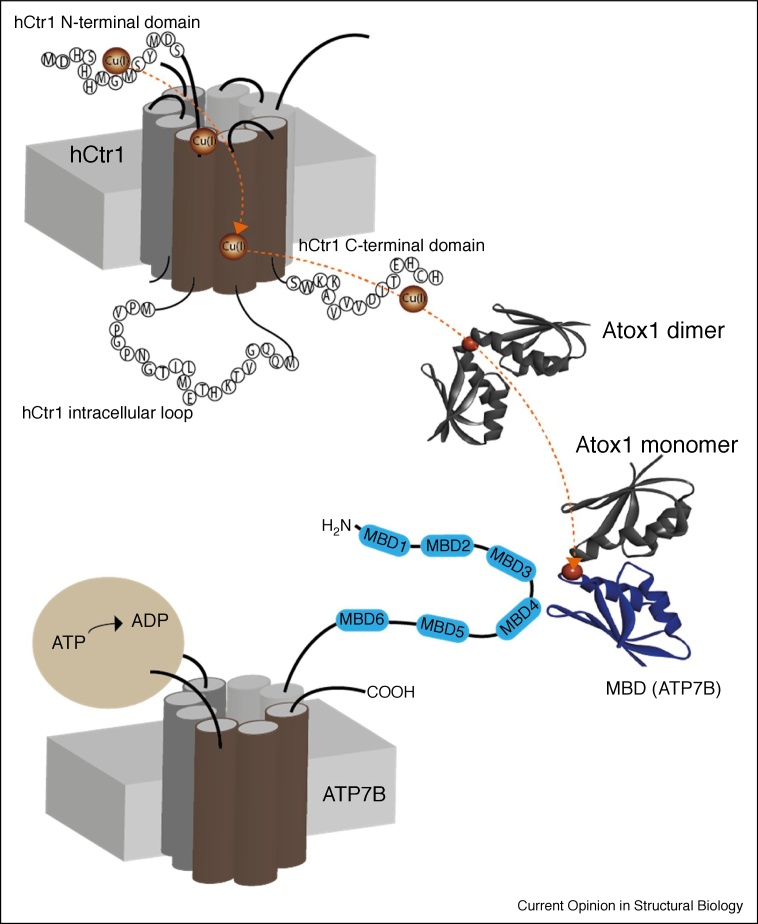
hCtr1-Atox1-ATP7B Cu(I) transfer model. Atox1 plays a critical role in mediating Cu(I) transfer mechanism, cycling between a dimer to monomer state and adjusting a specific conformation based on its target protein.

Atox1 interacts with the N-terminal domain of ATP7B, which contains six MBDs connected by linkers. Similar to the Atox1 metallochaperone, each MBD has a ferredoxin-like fold with a compact βαββαβ structure and a conserved metal-binding motif MxCxxC, located in the solvent-exposed β1-α1 loop. On the basis of the structures of Atox1 and of an MBD of ATP7B, Wernimont *et al.* proposed a mechanism for Cu(I) transfer from Atox1 to the MBD (Figure 3). In this model Atox1 transfers Cu(I) to a single MBD through consecutive ligand exchange reactions [44]. Subsequent studies used biophysical and computational methods to characterize the interactions between Atox1 and the six MBDs of ATP7A/B, focusing mostly on MBD4. NMR studies showed that the six MBD units (MBD1–6) can be differentiated into two groups, comprising MBD1–3, and MBD5–6, with MBD4 serving as a linker between them [14,44,52]. The structures of MBD3 and MBD4 were also solved using NMR [15,53]. Of all the MBDs, Atox1 interacts most strongly with MBD1-MBD4, whereas it does not interact with MBD5 or with MBD6 [14,16,54,55]. Cu(I) binding to regulatory MBD1–4 stimulates its transport by ATP7B and presumably facilitates the trafficking of the transporter by exposing sites for further modifications and protein–protein interactions [15,56^••^]. However, NMR studies and MD simulations revealed an exclusive interaction between Atox1 and MBD4, and not with MBD3 [19,54]. Whereas, smFRET experiments identified a dynamic situation in which, because of its structural flexibility, Atox1 can coordinate either MBD3, MBD4, or the two domains simultaneously, MBD3–4 [17^••^]. A recent study by our group, relying on a combination of EPR and MD simulations, revealed that Cu(I) extrusion is most likely mediated by Atox1 binding to MBD4 via the formation of transient interactions mediated by electrostatic complementarity of the two surfaces. Regarding the chemical mechanism, Cu(I) appears to be transferred by ligand exchange from C12/C15 of Atox1 to C370/C373 of MBD4 via the formation of several intermediates displaying a three-coordinated Cu(I) site (Figure 3). QM/MM simulations suggest that K60 of Atox1 may actively modulate the Cu(I) exchange [54]. All these experiments indicate that Atox1 interacts with MBDs in a monomeric state [57,58], in contrast to its interaction with hCtr1, as a dimer (Figure 3). Figure 4 shows a mechanistic picture of Cu(I) transfer from hCtr1 to MBD of ATP7B based on the current knowledge.

## Conclusions

In recent years, significant progress has been made toward elucidating the mechanisms underlying intracellular copper regulation. Multiple distinct studies have consistently revealed that the methionine segments and cysteine-based motifs present in the various components of the hCtr1-Atox1-ATP7A/B cycle are critical for Cu(I) binding. Studies investigating the effect of pH on Cu(I) affinity to proteins have provided critical insights regarding the possible mechanisms of cysteine ligand exchange reactions underlying metal transport. Discoveries regarding the conformational flexibility of Atox1, together with its capacity to function either in a dimeric or monomeric form, depending on its protein partner, indicate how the metallochaperone elegantly mediates Cu(I) transfer from hCtr1 to the Golgi apparatus and/or to its excretion route.

In spite of these achievements, many gaps remain in our understanding of this important metabolic pathway. In particular, knowledge of the mechanisms underlying hCtr1 and ATP7A/B function remains fragmentary, owing to the challenges involved in expressing and purifying these proteins for biophysical research, and in resolving a structural model at atomic-level resolution. It is necessary to overcome these obstacles, using a combination of biophysical, biochemical, and computational approaches, in order to identify all Cu(I) binding sites, and to obtain a comprehensive understanding of the Cu(I) transfer mechanism. As discussed above, current experimental and computational data suggest that each MBD in ATP7B has a specific role; these observations should be further analyzed and confirmed on the full-length ATP7B.

A more complete understanding of hCtr1-Atox1-ATP7A/B cycle may contribute toward the development of treatment for diseases associated with Cu-metabolism dysfunction, including Menkes’ and Wilson’s diseases. Current treatment for these conditions relies on Cu-chelators, which have led to an increased lifespan in some patients, yet are not uniformly effective. Moreover, although numerous studies have identified connections between copper regulation and neurological diseases such as Parkinson’s, Alzheimer’s and prion diseases, the underlying mechanisms of these connections remains elusive. Similarly, the role of Cu-transport and homeostasis in cancer, and in resistance to commonly used metal-based anticancer drugs, remains unresolved [9,10].

Herein, we have sought to depict current understanding of the Cu-transfer mechanism at the atomic level, emphasizing the delicate balance of transient protein–protein interactions underlying metal transfer, reactivity, and homeostasis. It remains a daunting challenge to harness this knowledge for future innovative therapeutic approaches aiming at counteracting the many pathological states associated with Cu-dis-homeostasis.

## Conflict of interest statement

Nothing declared.

## References and recommended reading

Papers of particular interest, published within the period of review, have been highlighted as:

• of special interest
•• of outstanding interest
